# Supplementary material for: Direct observation of polymer surface mobility via nanoparticle vibrations
Source: Nat Commun. 2018 Jul 25;9:2918. doi: 10.1038/s41467-018-04854-w (PMC6060150; doi:10.1038/s41467-018-04854-w)
Supplement: Supplementary file 1 — Supplementary Information [file 41467_2018_4854_MOESM1_ESM.pdf]

# Supplementary Information for:

# Direct Observation of Polymer Surface Mobility *via* Nanoparticle Vibrations

Hojin Kim[1], Yu Cang[2], Eunsoo Kang[2], Bartlomiej Graczykowski[2], Maria Secchi[3], Maurizio Montagna[4], Rodney D. Priestley[5], Eric M. Furst[1], George Fytas[2,6]

[1]*Department of Chemical and Biomolecular Engineering, University of Delaware, Newark, DE 19716, United States*

[2]*Max Planck Institute for Polymer Research, Ackermannweg 10, 55128, Mainz, Germany*

[3]*Department of Industrial Engineering, University of Trento, 38123 Trento, Italy*

[4]*Department of Physics, University of Trento, 38123 Trento, Italy*

[5]*Department of Chemical and Biological Engineering, Princeton University, Princeton, NJ 08544, United States*

[6]*IESL-FORTH, N. Plastira 100, 70013, Heraklion, Crete, Greece*

**Supplementary Note 1**

The study reveals the presence of a surface mobile layer from the temperature dependence of both interaction (s,1,1) mode and the inherent (s,1,2) mode, which additionally is split due to the interactions. From the qualitative calculations, we can conclude that the observed frequency blue-shift above $T_s$ results from the increase of the contact area radius, $a_0$, of several nanometers. However, it is difficult to make any quantitative statement how this increase relates to the thickness of mobile layer.

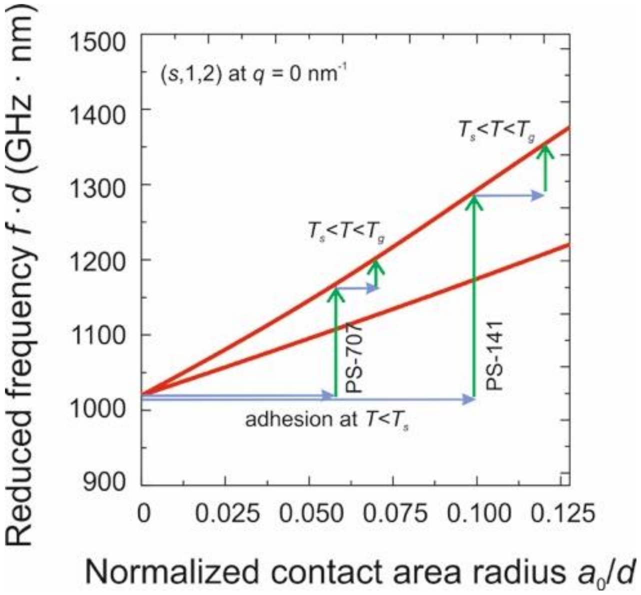


**Supplementary Figure 1**. Graphical presentation of enhanced resolution with decreasing NP size. Reduced frequency *f*(s,1,2)·*d* as a function of the normalized contact area radius calculated at Γ point (Supplementary Figure 9). The two branches (upper triplet and lower doublet) originating in (s,1,2) are presented. Horizontal arrows (long gray) depict the increase of the normalized contact area in the aggregation of the particles al low temperature, while the vertical arrows (green, shown only fir the upper triplet) indicate the corresponding blue-shift. Increasing temperature above $T_s$ (short gray arrow) leads to larger blue shifts (short green arrows) for smaller NP's (PS-141 vs PS-707)

Supplementary Figure 1 shows results of FEM calculations for fcc cluster of PS nanoparticles. This figure focuses only on the split and blue shift of the (s,1,2) (calculated at $q$=0 nm$^{-1}$) mode shown in a normalized presentation, *f*(s,1,2)·*d* vs $a_0/d$ (normalized contact are radius). For any particle diameter, d, the increase of the particle-particle contact results in: (i) mode splitting and (ii) blue-shift. Nevertheless, the magnitude

of these two effects depends on the particle size as we illustrate for d=141 nm (PS-141) and d=707 nm (PS-707). Following the JKR relation, $a_0 \propto d^{2/3}$ we take $a_0$=14 nm for PS-141 and $a_0$=41 nm for PS-707. (The numbers are arbitrary but provide the qualitative explanation.) Since $a_0/d \propto d^{-1/3}$, both (i) and (ii) interaction effects are larger for the smaller particle. This difference is even more apparent in the BLS original spectra (where the reduced frequency has to be divided by the particle diameter). Therefore, the mode splitting and the blue shift is less discernible for particles of bigger diameters and the temperature at which the mobile layer starts playing a role may change with surface to volume ratio of the particle. However, as the interactions give birth of (s,1,1) mode, $T_s$ is directly discernible in the temperature dependence of the f(s,1,1).

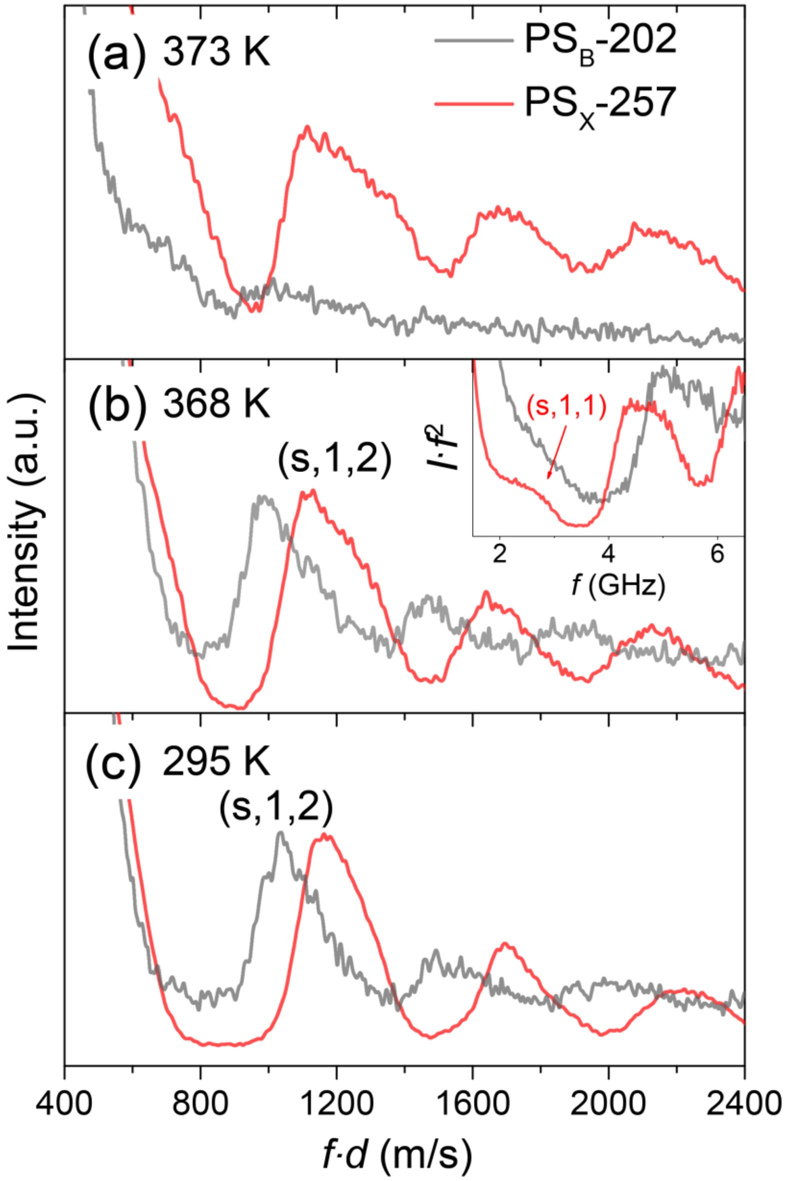


**Supplementary Figure 2**. The influence of crosslinking on the colloid vibration. Brillouin light scattering (BLS) spectra of two polystyrene spherical particles with diameter *d*=202 nm ($PS_B$-202, gray) and 257 nm ($PS_X$-257, red) in two presentations: *I(f)* vs. $f \cdot d$ (main plot) at **(a)** 295 K, **(b)** 368 K, and **(c)** 373 K and (inset to **(b)**) power spectra, $I \cdot f^2$ vs. $f$.

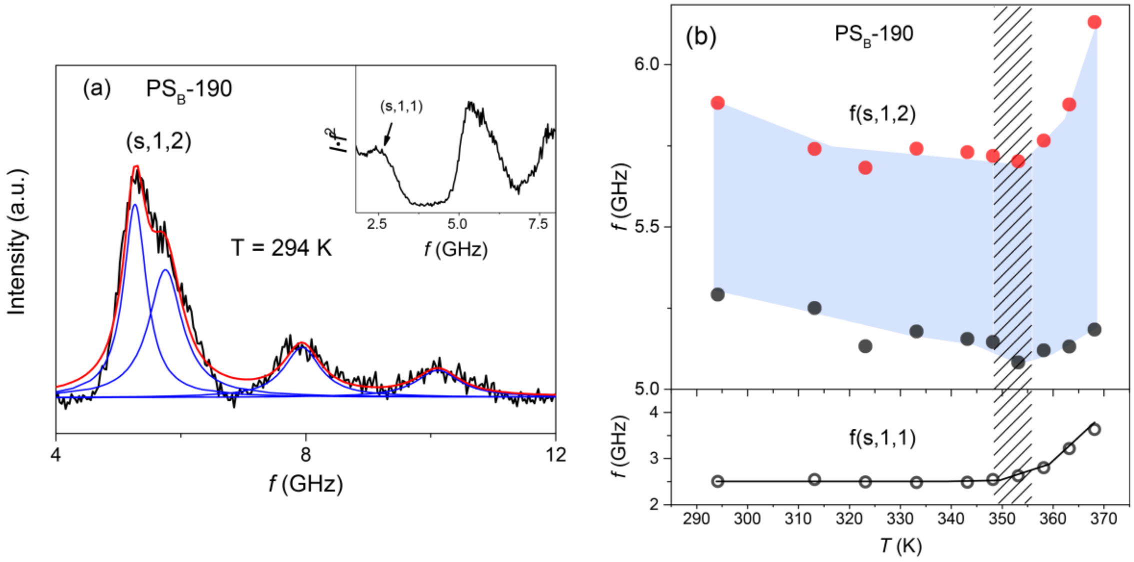


**Supplementary Figure 3**. Temperature dependence of $PS_B$-190 nanoparticle eigenfrequencies. **(a)** Brillouin light scattering (BLS) spectra of $PS_B$-190 particles at room temperature, T=293 K, as frequency $f$ vs. intensity, $I$. Inset: Reduced spectra $I \cdot f^2 \; vs. f$. Blue and red lines in **(a)** refer to Lorentzian lines of (1,2) mode and sum of them, respectively. **(b)** The temperature dependent $f$(1,1) and $f$(1,2) obtained at the maximum intensity for $PS_B$-190 are presented in the lower (open circles) and top panel (closed black and red circles indicating two frequencies of Lorentzian peaks), respectively. The hatched area in **(b)** indicates the softening $T_s$.

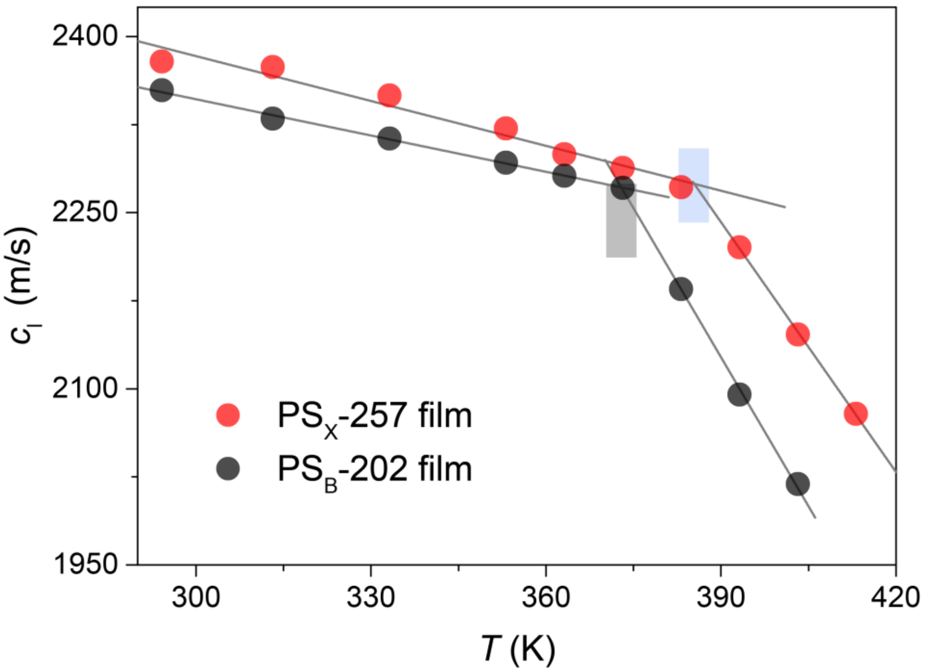


**Supplementary Figure 4.** Temperature dependence of longitudinal sound velocities in annealed bulk films. The longitudinal sound velocities ($c_l$) in annealed PS$_B$-202 (black) and PS$_X$-257 (red) bulk films are recorded by increasing temperature from room temperature, T=293 K. Black and blue filled areas refer to the $T_g$ of annealed PS$_B$-202 and PS$_X$-257 bulk films, respectively.

**Temperature dependent eigenfrequencies of core/shell nanoparticles**

As described in the "Discussion", the lack of $T_g$ depression can be attributed to a higher presence of NaVBS and AA at the surface. Therefore, given the fact that the surface mobility is due to either a gradient in dynamics (at near constant density) or a gradient in density, there should not be a lower density at the free surface for the neat PS NPs, which do not contain crosslinked networks. To support the latter, the temperature dependent eigenfrequencies of core/shell NPs (CS-168) consisting of a PS$_A$-141 core and a 14 nm shell are investigated. The shell is copolymer of styrene and trimethoxysilylpropylacrylate (TMSPA) with 10:1 styrene to TMSPA weight ratio. The density of this particle can be presumed to be very similar to PS.[6]

Based on Supplementary Figure 5, the CS-168 NP with homogeneously dense shell displays higher $T_s$ (by about 20K) and higher $T_g$ (by about 7K) than its parent $PS_A$-141 NP core. This corroborates the notion that the surface mobility is not the consequence of a gradient in density in the present polymer NPs. Instead, changing the surface dynamics in CS-168 NP impacts the softening transition temperature. Nevertheless, future work will systemically explore the role of surface capping and crosslinking on the observed properties of the nanoparticles.

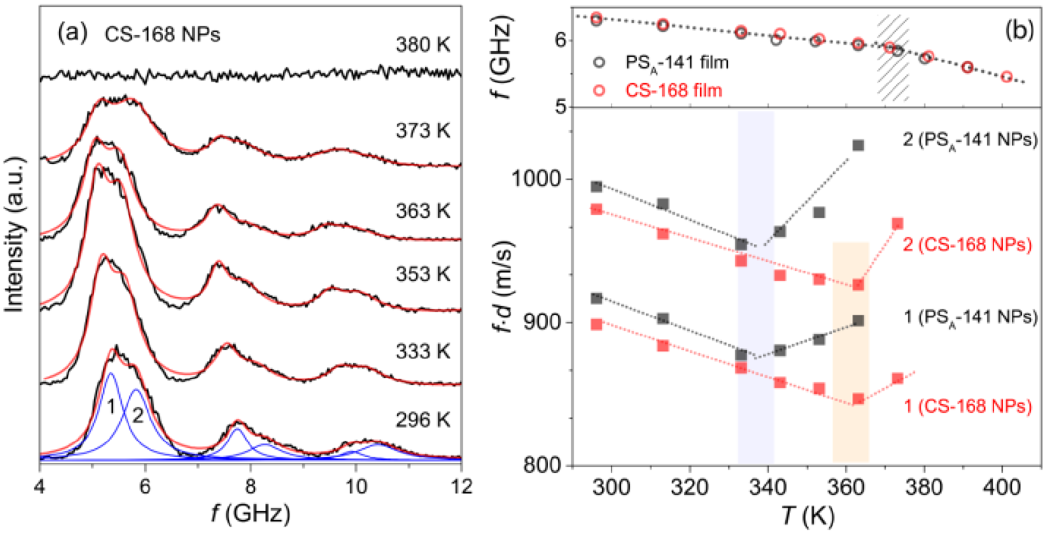


**Supplementary Figure 5**. Glass transition temperature and softening temperature of core-shell NPs with $PS_A$-141 core and PS- like thin shell. (a) BLS spectra at different temperatures represented by Lorentzian curves and the featureless BLS spectrum at 380 K indicating the formation of a contiguous film; this temperature is higher than that measured for the $PS_A$-141 of Fig.4a. (b) Temperature dependence of the frequency, $f$(s,1,1) of the interaction mode and the split (s,1,2) mode (lower panel) in CS-168 and $PS_A$-141 NP in an scaled version ($f$(s,1,2)·$d$) (lower panel) and the frequency of the longitudinal phonon, f (at q=0.0167 $nm^{-1}$) in the contiguous film obtained from the heating of CS-168 at 410 K (upper panel).

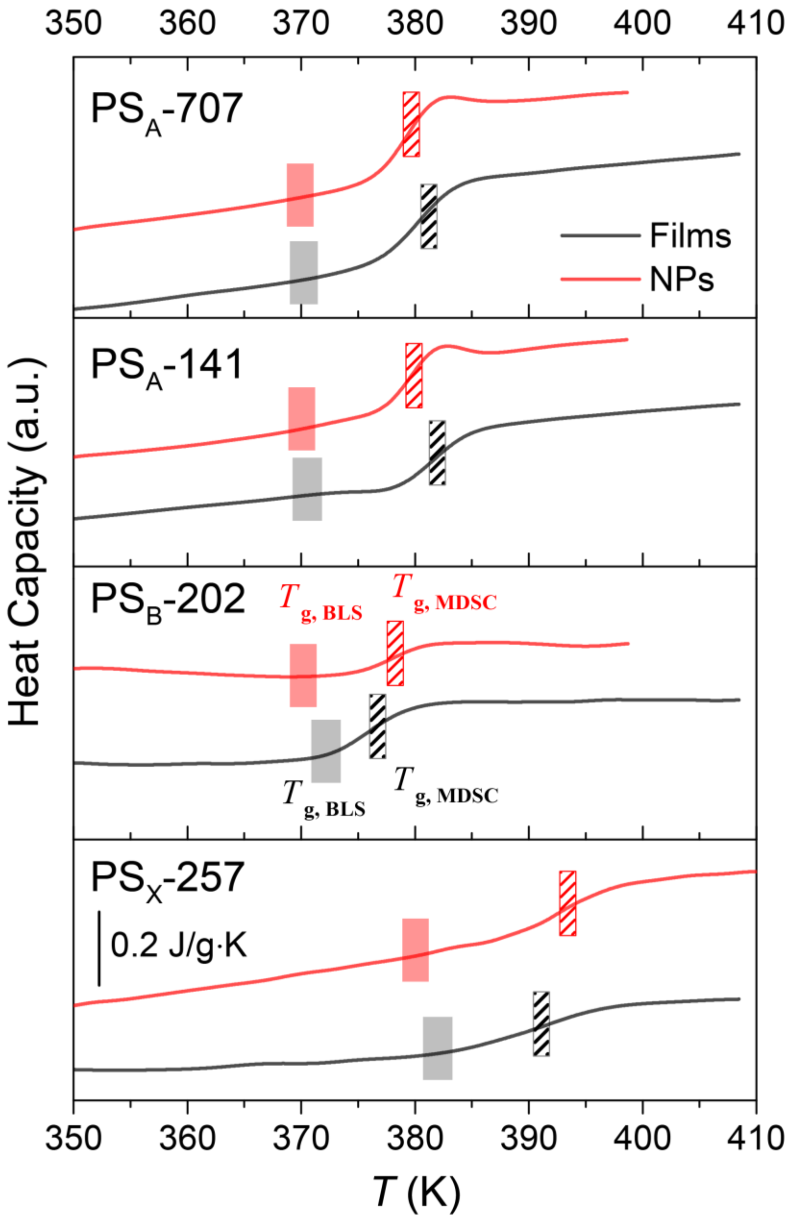


**Supplementary Figure 6.** The modulated differential scanning calorimetry (MDSC) thermograms plotted heat capacity (arbitrary unit) vs. temperature (K) both for NPs powder state (red solid line) and for annealed bulk state (black solid line) of $PS_A$-707, $PS_A$-141, $PS_B$-202, and $PS_X$-257 (from top to bottom). The filled and dashed areas indicate $T_g$ from BLS and from MDSC, respectively. The red and black areas refer to the $T_g$ of the NP cluster and that of annealed bulk polystyrene, respectively.

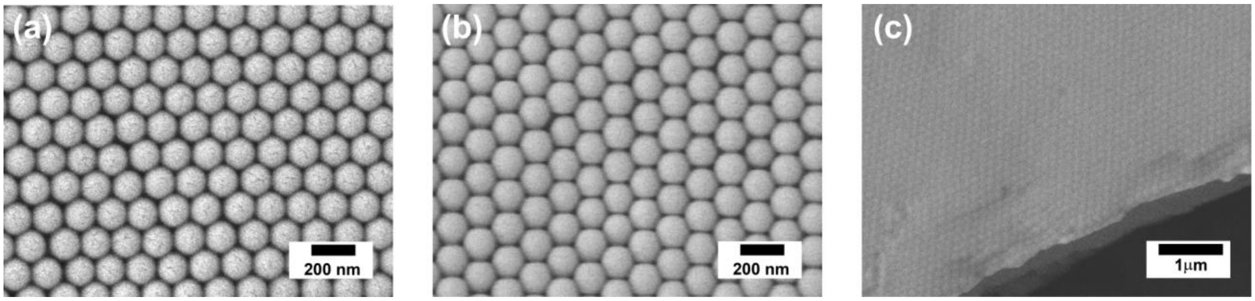


**Supplementary Figure 7.** Scanning electron microscope (SEM) images of $PS_A$-141 at room temperature **(a)** without thermal annealing, **(b)** after annealing at T=363 K ($T_g$>$T$>$T_s$), and **(c)** after annealing at $T$=383 K ($T$>$T_g$). Thermal annealing has been processed for 24 hrs.

## Calculation of BLS spectra

The mechanical eigenmodes of single polystyrene nanoparticles (PS-NPs) and the phonon dispersion in PS NPs arranged in fcc lattice were calculated using finite element method (COMSOL Multiphysics). The used material properties were: longitudinal speed of sound $c_\mathrm{l} = 2350\ \mathrm{m/s}$, transverse speed of sound $c_\mathrm{t} = 1210\ \mathrm{m/s}$, and mass density $\rho = 1050\ \mathrm{kg/m^3}$. The primitive unit cell is defined as shown in Supplementary Figure 8. For simplicity we assume perfect bonding among the neighboring NPs. This interaction is governed by the circular contact area of radius $a_0$, which follows Johnson-Kendall-Roberts (JKR) model. In this case, $a_0$ can be related to the NP diameter $d$ as following: $a_0 \cong 0.24\ d^{2/3}$. Using the notation as in Supplementary Figure 8, the primitive basis vectors are $\mathbf{a_1} = \frac{a'}{2}[0,1,1]$, $\mathbf{a_2} = \frac{a'}{2}[1,0,1]$, and $\mathbf{a_3} = \frac{a'}{2}[1,1,0]$, where $a' = \sqrt{2(d^2 - 4a_0^2)}$ is the lattice reduced by the particle adhesion. The basis vectors of the reciprocal lattice are further defined in a common manner. To check the effect of the increased contact area we calculated eigenfrequecies at $\Gamma$ ($\mathbf{q} = [0,0,0]$) for d=141 nm (for example, $PS_A$-141) as a function of $a_0$ . The results and the normalized values (top and right axis) are displayed in Supplementary Figure 9. Since only spheroidal modes are BLS active, we may conclude that A1-C1 and L1-P1 modes are in principle observed in BLS. A1-C1 have zero frequency at $q$ = 0 and non-zero for L1-P1. These modes (L1-P1) are originated from (s,1,2) mode at $a_0 = 0$ and split into two groups, 1) L1 and M1 and 2) N1, O1, and P1. At

higher $a_0$, f(s,1,2) gradually increase. In other words a finite contact area results in two (s,1,2) peaks in BLS spectra, which can increase their frequency and splitting upon an increased contact area. Nevertheless, as the contact area radius scales as $a_0 \sim d^{2/3}$ this effect is less pronounced for bigger spheres. This is evident if we use normalized units (top and right axis in Supplementary Figure 9). Here we compare $PS_A$-141and $PS_A$-707 by taking $a_0$ based on JKR model; for $d = 707$ nm, $a_0 = 19.2$ nm and for $d = 141$ nm, $a_0 = 6.6$ nm. In this case (dotted arrows in Supplementary Figure 9) the expected normalized frequency and splitting is smaller for PS-707, what agrees with the BLS experimental data.

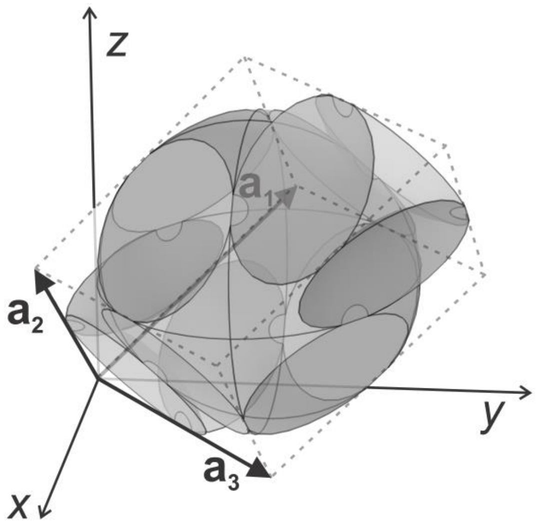


**Supplementary Figure 8.** Primitive unit cell of a particle with interaction among neighboring particles used for finite element method calculation.

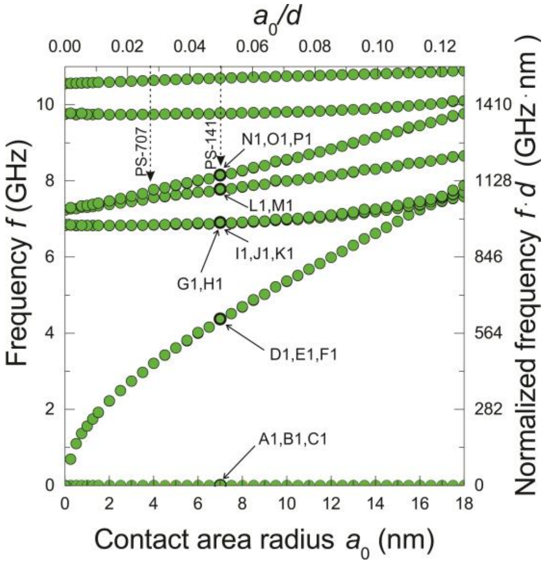


**Supplementary Figure 9.** Mechanical eigenmode frequencies as a function of radius $a_0$ the contact area, calculated for PS sphere with diameter, *d*=141 nm, at Γ ($q = [0,0,0]$).

**Brillouin spectra of fcc crystals of spheres**

The five (s,1,2) modes periodically transform the sphere in an oblate and a prolate ellipsoid. At **q**=0, where all particles move in phase, the quintet splits in a doublet and a triplet due to the lowering of the symmetry from spherical to cubic. Further degeneracy is removed at $\mathbf{q} \neq 0$. In principle, one could calculate the Brillouin spectra by the previous method,[1] if the mode pattern are available by FEM calculations. The following quantities are important: i) the maximum q-value in the experiment (in our case, $\mathbf{q}_{\mathrm{bs}} = 4\pi n/\lambda = 0.375\ \mathrm{nm}^{-1}$, meaning back scattering, for $\lambda = 532$ nm and a refractive index of PS, $n = 1.59$) and ii) the size of the Brillouin zone, measurable by the $\mathbf{q}_{\mathrm{X}}$ value at the X-point ($\mathbf{q}_{\mathbf{X}} = \pi/D_{100}$); the q-dependence of the Brillouin activity, that has a broad maximum at about $qd = 6.4$ in the free sphere. In the case of $\mathrm{PS_A}$-141 particle, $\mathbf{q}_{\mathrm{X}} = 0.032\ \mathrm{nm}^{-1}$, smaller than $q_{\mathrm{bs}}$. Therefore, Brillouin scattering can occur at all q-vectors of the Brillouin zone. This is quite different from the Brillouin spectroscopy by laser light in simple crystals, where the size of the unit cell is of the order of a fraction of nm, and the Brillouin zone (BZ) has a size of the order of $10\ \mathrm{nm}^{-1}$, and where only a small volume of the BZ around the Γ-point is achievable. It is similar to the Brillouin spectroscopy by inelastic X-ray scattering. For even larger sizes of

the unit cell, the BZ size is further reduced and many BZs are achievable. In the presence of multiple scattering, all q-vectors contribute. The lineshapes of the Brillouin bands will resemble the density of phonon states, but modulated by a q dependence of the activity.[1–4] Therefore, we will use only qualitative argumentation to describe the shape of the Brillouin bands.

The sound velocities can be estimated from the cut-off frequencies reported in Fig. 1c that is associated with the frequency of the longitudinal acoustic band at the X point of an fcc crystal. In fact, for phonon propagation along the [100] direction, the system should be well described by a linear chain of mass $M$ at distance $D_{100} = \sqrt{(d^2 - 4a_0^2)/2}$ between next planes, coupled by elastic springs of stiffness $K_{l,t}$, for longitudinal (subscript $l$) or transverse (subscript $t$) phonons. The frequency at the X-point is given by $\omega_{\mathrm{l,t}} = 2\left(K_{\mathrm{l,t}}/M\right)^{1/2}$ and the sound velocity by $c_{\mathrm{l,t}} = D_{100}\left(K_{\mathrm{l,t}}/M\right)^{1/2}$.[5] Therefore, $c_{\mathrm{l,t}} = D_{100}\omega_{\mathrm{l,t}}/2$. In this way, the sound velocities of the clusters can be estimated by taking the measured values of $\omega_{\mathrm{l}} = 2\pi f(1,1)$ from Fig. 1c. The estimated values are reported in Supplementary Table 1.

As for the (s,1,2) band, the FEM calculations show two main results: i) the phonon band has its low frequency tail at the Lamb frequency of the free particle; ii) a splitting at q=0 and a nearly flat dispersion of two components. The latter suggests a two peaked DOS, associated with the observed two peaked structure of the experimental (s,1,2) shapes of Fig. 1a. Based on the calculated BLS spectra, a rough estimation can be made using two maxima of the DOS ($f_1$ and $f_2$ indicating the lower and higher peak, respectively): $f_1 - f_2 \approx f_1 - f_{\mathrm{L}}(s,1,2)$, where $f_{\mathrm{L}}(\mathrm{s},1,2)$ is the frequency of the free sphere. Within this simple model, we have two methods for estimating the single particle frequency: i) $f_{\mathrm{L}}(s,1,2) = f_{\mathrm{lfco}}$ where $f_{\mathrm{lfco}}$ refers to the low frequency cutoff of the observed peak and ii) $f_{\mathrm{L}}(s,1,2) = 2f_1 - f_2$, when the splitting is observed. FEM calculations at $q$=0 as a function of $a_0$ (Supplementary Figure 9), which gives a measure of the strength of the interaction, show that the splitting and the shift of the doublet (L1 and M1) and triplet (N1, O1, and P1) of the (s,1,2) modes are nearly linear with $a_0$. This ensures that the above two methods can be used for particles having different sizes and interactions among them. In all cases when the two

methods are used, the same result is obtained within the estimated error and the calculation results are reported in Supplementary Table 1 together with the effective transverse sound velocity of the free particle obtained by inverting the Lamb equation (1).

**Supplementary Table 1. Estimated $f_{\mathrm{L}}(s,1,2)$ by the equation, $f_{\mathrm{L}}(s,1,2)=2f_1-f_2$ (values in the parenthesis are from the equation $f_L(s,1,2)=f_{lfco}$). $c_{t,eff}$ is obtained by using the Lamb equation in equation (1). For $f_{\mathrm{L}}(s,1,2)\cdot d$ and $c_{t,eff}$, an error of about 5% has been estimated on the basis of the used methods. The values for PS-707 is derived from a rough deconvolution of the experimental lineshape (a nearly Gaussian with a width that is about 90% of the width of the observed peak).**

| Sample | $f_1$ [GHz] | $f_2$ [GHz] | $f_{\mathrm{L}}(s,1,2)$ [GHz], (± 20) | $f_{\mathrm{L}}(s,1,2)\cdot d$ [m/s], (± 40) | $c_{t,eff}$ [m/s], (± 50) |
|---|---|---|---|---|---|
| $PS_A$-141 | 6.5 | 7.1 | 6.0 (6.0) | 840 (840) | 1000 |
| $PS_B$-190 | 5.3 | 5.9 | 4.7 (4.7) | 900 (900) | 1070 |
| $PS_B$-202 | 5.1 | 5.6 | 4.6 (4.6) | 930 (930) | 1100 |
| $PS_x$-257 | 4.5 | 4.9 | 4.1 (4.0) | 1040 (1020) | 1240 |
| $PS_A$-707 | - | - | - (1.2) | - (840) | 1000 |

**Supplementary References**


1. Montagna, M. Brillouin and Raman scattering from the acoustic vibrations of spherical particles with a size comparable to the wavelength of the light. *Phys. Rev. B - Condens. Matter Mater. Phys.* **77,** 1–9 (2008).
2. Still, T., Mattarelli, M., Kiefer, D., Fytas, G. & Montagna, M. Eigenvibrations of submicrometer colloidal spheres. *J. Phys. Chem. Lett.* **1,** 2440–2444 (2010).
3. Mattarelli, M., Montagna, M., Still, T., Schneider, D. & Fytas, G. Vibration spectroscopy of weakly interacting mesoscopic colloids. *Soft Matter* **8,** 4235 (2012).
4. Mattarelli, M., Secchi, M. & Montagna, M. Phononic crystals of spherical particles: A tight binding approach. *J. Chem. Phys.* **139,** (2013).
5. Ashcroft, N. & Mermin, N. *Solid State Physics*. (Saunders College, 1976).
6. Tissot, I. & Novat, C. Hybrid Latex Particles Coated with Silica. *Macromolecules* **34,** 5737–5739 (2001).
